# Effects of Akt Activator SC79 on Human M0 Macrophage Phagocytosis and Cytokine Production

**DOI:** 10.3390/cells13110902

**Published:** 2024-05-24

**Authors:** Robert J. Lee, Nithin D. Adappa, James N. Palmer

**Affiliations:** 1 Department of Otorhinolaryngology—Head and Neck Surgery, Perelman School of Medicine, University of Pennsylvania, Philadelphia, PA 19104, USA; nithin.adappa@pennmedicine.upenn.edu (N.D.A.); james.palmer@pennmedicine.upenn.edu (J.N.P.); 2 Department of Physiology, Perelman School of Medicine, University of Pennsylvania, Philadelphia, PA 19104, USA

**Keywords:** signal transduction, live cell imaging, monocyte-derived macrophages, inflammation, innate immunity, Toll-like receptor

## Abstract

Akt is an important kinase in metabolism. Akt also phosphorylates and activates endothelial and neuronal nitric oxide (NO) synthases (eNOS and nNOS, respectively) expressed in M0 (unpolarized) macrophages. We showed that e/nNOS NO production downstream of bitter taste receptors enhances macrophage phagocytosis. In airway epithelial cells, we also showed that the activation of Akt by a small molecule (SC79) enhances NO production and increases levels of nuclear Nrf2, which reduces IL-8 transcription during concomitant stimulation with Toll-like receptor (TLR) 5 agonist flagellin. We hypothesized that SC79’s production of NO in macrophages might likewise enhance phagocytosis and reduce the transcription of some pro-inflammatory cytokines. Using live cell imaging of fluorescent biosensors and indicator dyes, we found that SC79 induces Akt activation, NO production, and downstream cGMP production in primary human M0 macrophages. This was accompanied by a reduction in IL-6, IL-8, and IL-12 production during concomitant stimulation with bacterial lipopolysaccharide, an agonist of pattern recognition receptors including TLR4. Pharmacological inhibitors suggested that this effect was dependent on Akt and Nrf2. Together, these data suggest that several macrophage immune pathways are regulated by SC79 via Akt. A small-molecule Akt activator may be useful in some infection settings, warranting future in vivo studies.

## 1. Introduction

Macrophages are important players in both bacterial [1,2] and viral immunity [3,4] as well as cancer [5]. Understanding mechanisms of macrophage function may reveal novel targets for boosting immune responses to combat infections without the use of conventional antibiotics. Such strategies would be highly useful in the setting of upper respiratory infections [6]. For example, conventional management of upper respiratory infections, including acute and chronic rhinosinusitis, accounts for ~20% of adult antibiotic prescriptions [7], acting as a major diver of the ongoing crisis of antibiotic resistance, named by the World Economic Forum as “arguably the greatest risk…to human health” [8]. New therapies for respiratory and other infections are needed [9,10,11], which could be obtained via targeting host signaling pathways to stimulate endogenous innate immune defenses. 

The Akt family of kinases can phosphorylate multiple downstream targets involved in cell survival and/or proliferation [12], such as transcription factors or other downstream kinases that regulate metabolism [12]. Another important target of Akt in several cells is endothelial (e) and neuronal (n) nitric oxide synthases (eNOS and nNOS, respectively). While eNOS and nNOS were named based on their initially discovered roles in endothelial cells and neurons, these NOS isoforms are expressed in a diverse array of cell types [13] and acutely regulated by kinases and/or Ca^2+^/calmodulin binding [14,15]. For example, we extensively studied how eNOS in airway ciliated cells regulates ciliary beating and the innate defense process of mucociliary clearance [16]. 

We also previously showed that activation of bitter taste receptors (taste family 2 receptors or T2Rs) in unpolarized (M0) human macrophages stimulates Ca^2+^-dependent e/nNOS activation to enhance bacterial phagocytosis via the downstream production of cGMP [17,18,19]. Prior studies also showed that stimulation of Fcγ receptors activates e/nNOS to enhance phagocytosis in M0 macrophages [20]. However, few other studies have focused on macrophage e/nNOS signaling [13,20,21,22,23,24,25,26,27]. Most studies on macrophage NOS focus on inducible (i) NOS during macrophage activation and NFκB induction [28,29]. Our data suggest that defects in e/nNOS signaling in macrophages may potentially play a role in the early stages of respiratory infection in diseases like chronic rhinosinusitis and cystic fibrosis (CF) [30]. Impaired function of the CF transmembrane conductance regulator (CFTR) results in impaired T2R signaling to e/nNOS in human M0 macrophages [18]. While Ca^2+^ responses downstream of T2Rs were unaffected, the translation of these Ca^2+^ responses to e/nNOS activation was impaired, resulting in less NO production [18].

While e/nNOS are both activated by Ca^2+^/calmodulin binding, the AKT phosphorylation of human eNOS and nNOS can also activate NO production independently of Ca^2+^ [31,32,33,34], including in nasal and lung epithelial cells [35,36,37]. We hypothesized that one way to enhance macrophage phagocytosis in, for example, CF patient macrophages, might be to target Akt directly to enhance innate killing. Several studies have pointed toward defects in CF macrophage function [38,39,40], making this cell a potential target for CF respiratory infections. While some have reported altered receptor-dependent Akt signaling in CF cells [41,42,43], we did not observe alterations to Akt signaling in CF nasal epithelial cells using a small-molecule Akt activator [37], suggesting that this pathway may be intact and druggable in CF macrophages. Macrophages are important players in early airway innate immunity [44,45], and enhancing macrophage function may help combat infections in CF. 

Here, we tested the effects of direct Akt activation in human macrophages using SC79, a compound that interacts with Akt to change its conformation and allow the phosphorylation of Akt by upstream kinases [46,47,48,49,50]. SC79 has been shown to have beneficial effects in some animal models of hepatic [50] and neuronal [48,49] disease, linked to SC79’s activation of Akt-driven survival pathways. We previously demonstrated that SC79 acutely increased Akt’s phosphorylation at one of its activating sites (S473) in multiple types of lung and nasal epithelial cells [35]. The resulting Akt activation and NO production from these epithelial cells was bactericidal against the common respiratory opportunistic pathogen, *Pseudomonas aeruginosa* [37]. We hypothesized that the activation of Akt-dependent NO production in human macrophages may enhance bacterial phagocytosis, like the NO activated downstream of T2Rs. 

Moreover, we also previously observed that SC79 caused the translocation of an important anti-oxidant transcription factor, Nrf2, to the nucleus in nasal and lung epithelial cells [35]. Nrf2 is normally restricted from the nucleus via its interaction with KEAP-1 that targets Nrf2 for ubiquitination and degradation [51,52]. Activation of Akt has been shown to enhance Nrf2 activity via downstream phosphorylation and the inhibition of glycogen synthase kinase-3beta (GSK-3β) [53]. GSK-3β phosphorylation of Nrf2 contributes to Nrf2 targeting for degradation [54,55], and thus the Akt phosphorylation and inhibition of GSK-3β enhance Nrf2 protein stability [56,57] and increase the transcription of Nrf2 targets [58]. Nrf2 activates the transcription of genes containing anti-oxidant response elements (AREs) [59]. However, in many cases, Nrf2 can also reduce the expression of many NFκB gene targets [60,61,62]. We found that the SC79 induction of Nrf2 in lung and nasal epithelial cells reduces the transcription of NFκB-driven IL-8 (CXCL8) downstream of Toll-like receptor (TLR) and TNFα receptor activation [35]. IL-8 is an important chemotactic and inflammatory cytokine/chemokine in inflammation all over the body [63,64], and a reduction in IL-8 suggests the effect of less inflammation, so SC79 may be anti-inflammatory in certain settings. Thus, here, we also tested if/how SC79 affects macrophage cytokine responses to bacterial lipopolysaccharide (LPS), an important activator of TLR4 and other pathogen recognition receptors [65,66] produced by Gram-negative bacteria like *P. aeruginosa* [67,68]. 

## 2. Materials and Methods

### 2.1. Cell Culture and General Reagents

Primary human unprimed (M0) macrophages were cultured as described [17,18,19] in RPMI1640 media with 10% human serum and penicillin/streptomycin for cell culture at 1× concentration (Gibco/ThermoFisher Scientific, Waltham, MA, USA). De-identified purified monocytes from healthy apheresis donors were obtained from the University of Pennsylvania Human Immunology core with the written informed consent of every participant and institutional review board approval. Monocytes were isolated via RosetteSep Immunodensity cell separation (StemCell Technologies, Vancouver, BC, Canada). Cells isolated from 10 different individuals were used. As all samples were de-identified in terms of race, age, sex, etc., the samples were used in a blinded fashion. Macrophages were differentiated by adherence culture for 12 days in 8-well chamber slides (CellVis; Mountain View, CA, USA), as described [17]. Our prior studies [17,18,19] suggest no differences in T2R or phagocytosis responses among macrophages differentiated by adherence alone or by adherence plus M-CSF [17]. Others have also shown differentiation of macrophages in the absence of M-CSF [69,70,71], and thus only adherence was used for these studies. For all experiments, macrophages collected on different days from at least 3 different donors were tested. 

LDH assay kit was from Abcam (Cambridge, MA, USA). *P. aeruginosa* LPS was from Sigma (St. Louis, MO, USA). GSK690693, H89, U73343, U73122, LY294002, MK2206, cPTIO, D-NAME, L-NAME, SNAP, and SC79 were from Cayman Chemical (Ann Arbor, MI, USA). All other reagents were from MilliporeSigma (St. Louis, MO, USA). Stock solutions of hydrophobic compounds were made at ≥1000× in DMSO. The final concentration of DMSO in working solutions was ≤0.1% with the control containing the highest DMSO concentration used in the individual experiment types. Pharmacological inhibitors and targets are in Table 1. 

ELISAs for TNFα, IL6, and IL12 were from Peprotech (Cranbury, NJ, USA), and IL8 ELISA was from ThermoFisher Scientific. All ELISAs were carried out in accordance with the manufacturers’ instructions, as described previously [72,73].

*P. aeruginosa* conditioned media were obtained from *P. aeruginosa* lab strains PAO-1 (ATCC 15692), and we used clinical CRS-isolates P11006, 2338, and L3847 (obtained from Drs. N. Cohen and L. Chandler, Philadelphia VA Medical Center [74]). Colonies were grown overnight to OD ~1 in LB media (Gibco), followed by resuspension in RPMI 1640 media at an OD of 0.1. Bacteria were grown overnight (~18 h) in RPMI 1640. Bacteria were then removed via centrifugation followed by filtration through a 0.2 µm filter. Conditioned bacteria media were used at a 25% concentration diluted in unconditioned RPMI 1640 media (no FBS) for cytokine production experiments. Mock-conditioned RPMI 1640 (overnight incubation with no bacteria) was used as a control. All cytokine production/release/transcription experiments were carried out with overnight incubation in serum-free RPMI 1640 media. 

### 2.2. Quantitative PCR (qPCR)

RNA was isolated from macrophages as described previously [17,75] (RNA extraction kit, Zymo Research; Irvine, CA, USA). Quantitative (q) PCR was performed using TaqMan primer assays (ThermoFisher) for indicated genes and UBC as a housekeeping gene in separate reactions. Relative expression in experiments with control conditions was calculated using the 2^−ΔΔCt^ method while experiments reporting expression vs. UBC utilized the 2^−ΔCt^ method. The primers used in this study have been described previously [17,35,76,77]. 

### 2.3. Immunofluorescence (IF) Microscopy

IF microscopy was carried out as previously described [17,18,19,78]. Macrophages on a chambered coverglass (CellVis, Mountain View, CA, USA) were fixed in 4% formaldehyde (20 min, room temperature) followed by permeabilization and blocking in Dulbecco’s phosphate-buffered saline (DPBS) plus 1% bovine serum albumin (BSA), 5% normal donkey serum (NDS), 0.2% saponin, and 0.1% triton X-100 at 4 °C for one hour. After washing with DPBS, samples were incubated with primary antibody (1:100; 4 °C; overnight). After further washing, samples were incubated with AlexaFluor (AF)-labeled secondary antibodies (1:1000; 4 °C; 2 h; donkey anti-rabbit AlexaFluor 546, donkey anti-mouse AlexaFluor 488, or donkey anti-goat AlexaFluor 647; ThermoFisher). Samples were imaged after further washing and the addition of mounting media (Fluoroshield with DAPI; Abcam; Cambridge, MA, USA). Imaging was performed on an Olympus (Tokyo, Japan) IX-83 microscope, with a 60× (1.4 NA) objective, and a Hamamatsu Orca Flash 4.0 camera (Tokyo, Japan), using Metamorph (Molecular Devices, San Jose, CA, USA). Raw images were analyzed in FIJI (version 2.14) [79] using only linear adjustments (min/max) and no gain or gamma settings. Antibody for αtubulin was from Developmental Studies Hybridoma Bank (12G10; mouse monoclonal), antibody for Akt was from Cell Signaling Technologies (9272S; rabbit polyclonal), and antibody for eNOS was from Santa Cruz (sc-12972; goat polyclonal).

For the expression of macrophage markers, fixed macrophages were stained for markers shown to be expressed in M0 human monocyte-derived macrophages [80,81,82,83], including CD14 (conjugated to FITC; mouse monoclonal clone MEM-15; ThermoFisher Scientific/Invitrogen, Waltham, MA USA MA1-19592), CD16 (conjugated to FITC; mouse monoclonal clone 3G8; MA5-44096 and CD68 (conjugated to FITC; clone KP1; Novus NB100683F), or the isotype control (normal mouse IgG1 conjugated to FITC; Abcam ab91356). Imaging cytometry was performed using fluorescence microscopy as described [17]. Chamber wells were scanned with a 10× (0.4 NA; PlanApo) objective using the Metamorph slide scanner function on an Olympus IX83 microscope and a standard FITC filter set. Intensities of individual cells were automatically determined by the integrated morphometry plugin. Cell detection was validated by comparing cell counts with DAPI stain nucleus counts, and was found to differ by ≤2%. Approximately 2000–3000 cells were counted per well. 

### 2.4. Live Cell Imaging of Akt Activation

Macrophages on 8-well chambered coverglass were transfected the next day with Effectene (Qiagen, Germantown, MD, USA) and the FRET-based ratiometric sensor AktAR (obtained from Jin Zhang via Addgene, Watertown, MA, USA [84]). Imaging was performed on an inverted microscope, the Olympus IL83 microscope, a with 20× (0.8 NA) objective, CFP/YFP filters (Chroma Technologies, Bellows Falls, VT, USA), and Lambda LS filter wheels (Sutter, Novato, CA, USA). Images were acquired using MetaFluor (Molecular Devices). SC79, SNAP, L-NAME, D-NAME, cPTIO, MK2206, LY294002, U73122, U73343, GSK690693, and colistin sulfate were from Cayman Chemical (Ann Arbor, MI, USA).

### 2.5. Live Cell Imaging of Calcium, NO, and cGMP Production

Macrophages on glass chambered coverslips (CellVis) were loaded with 5 µM Fluo-4-AM (to measure calcium) or 5 µM DAF-FM diacetate (to measure NO) for 45 min as previously described [17,85] in 20 mM HEPES-buffered Hank’s Balanced Salt Solution (HBSS; pH 7.4) with 1.8 mM Ca^2+^. Either Fluo-4-loaded or DAF-FM-loaded cells were imaged as previously described [18,19,78] on a TS100 microscope (Nikon, Tokyo, Japan) with a 20× 0.8 NA PlanApo objective, Retiga R1 CCD camera (Photometrics, Tucson, AZ, USA), standard FITC/GFP filter set (Chroma, Bellows Falls, VT, USA), and XCite 110 LED illumination source used as an instant on/off shutter. Images were acquired and analyzed with Micromanager ImageJ (Version 2.0) [86] and FIJI (Version 2.14) [79].

For the Green GENIe cGMP biosensor (Montana Molecular, Bozeman, MT, USA), macrophages were transfected with mammalian-modified baculovirus (BacMam; *Autographa californica* pseudotyped to infect mammalian cells [87,88,89]), as previously described [17]. Imaging was carried out using the same settings described above.

### 2.6. Phagocytosis Assays

Phagocytosis assays were performed as described [17]. Macrophages were incubated in phenol red-free, low-glucose DMEM with heat-killed FITC-labeled *Escherichia coli* at 250 µg/mL (strain K-12; reagents from Vybrant phagocytosis assay kit; ThermoFisher; cat # E2861) ± SC79 for 15 min at 37 °C. Extracellular FITC was quenched with trypan blue, and fluorescence was recorded on a Spark 10M plate reader (Tecan, Männedorf, Switzerland; 485 nm excitation, 535 nm emission). As phagocytosis is negligible at 4 °C up to room temperature [17], we recorded the fluorescence from living cells at room temperature immediately after the FITC-*E. coli* incubation. For the representative micrograph shown, macrophages on glass were incubated as above, extracellular FITC was quenched with trypan blue, and cells were washed ≥5× in PBS to remove residual extracellular FITC-*E. coli*. Remaining adherent MΦs were fixed in 4% formaldehyde (Electron Microscopy Sciences, Hatfield, PA, USA) for 10 min followed by being subject to DAPI staining in mounting media (Fluoroshield with DAPI, Abcam). *E. coli* were then imaged using standard FITC filters (Semrock, Rochester, NY, USA) on an inverted Olympus IX-83 microscope with a 20× (0.8 NA) objective, XCite 120LEDBoost illumination, and a Hammamatsu (Tokyo, Japan) Orca Flash 4.0 camera. 

Phagocytosis assays were also carried out similarly using 125 µg/mL pHrodo red-labeled *S. aureus* (strain Wood 46; ThermoFisher, cat #A10010) [17]. As pHrodo dyes only fluoresce when particles are internalized into low-pH endosomes (previously tested by our own group [17]), this assay does not require washing or quenching of the extracellular pHrodo *S. aureus*. Macrophages were incubated with pHrodo-*S. aureus* for 30 min at 37 °C as described [17] with excitation at 555 nm and emission at 595 nm measured on the Tecan Spark 10 M plate reader. Background measurements were made using wells containing fluorescent *S. aureus* in the absence of macrophages. Representative micrograph images were taken as above except using a standard TRITC filter set (Semrock). The images shown for comparison were collected on the same day under identical conditions with identical min/max settings. No non-linear (e.g., gamma) adjustments were made. For superoxide experiments, macrophages were loaded with 2.5 µM MitoSox or dihydroethidium for 45 min at room temperature, with subsequent incubations carried out at 37 °C. 

### 2.7. Statistical Methods

Numerical data were compiled and analyzed in Excel (Version 16.85, Microsoft, Redmond, WA, USA) and/or Prism (Version 10, GraphPad software, La Jolla, CA, USA). One-way ANOVA and a Bonferroni’s post-test (pre-selected pairwise comparisons) or Dunnett’s post-test (for comparing the result to the control value) were used for multiple comparisons. Asterisks (*) or pound signs (#) were used to denote *p* < 0.05, the cutoff for statistical significance for this study. Bar graphs show both the mean ± SEM and individual data points derived from the biological replicates (i.e., separate experiments conducted with different donor cells on different days). All data generated during this study were analyzed and are shown in this article. Raw numerical data points from traces or bar graphs shown here are available upon request. 

## 3. Results

### 3.1. Akt Expression and Function in Human Monocyte-Derived Macrophages

**Figure 1:**
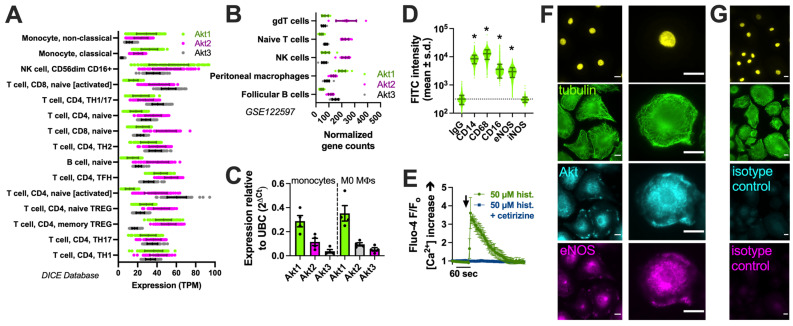
Akt isoform expression in M0 macrophages. (**A**) Expression (transcripts per million, TPM) of Akt isoforms in immune cells in the DIC database; (**B**) Normalized gene counts of Akt isoforms from GEO dataset GSE122597. (**C**) Expression of Akt isoforms relative to housekeeping gene UBC determined via performing qPCR on the monocytes and derived M0 macrophages used here; data points show results from 3 independent donors. (**D**) Imaging cytometry analysis of staining of macrophage markers (CD14, CD68, and CD16) with eNOS and iNOS in M0 macrophages. All markers were significantly above the control (mouse IgG) except iNOS, which was upregulated in M1 macrophages; * *p* < 0.05 vs. IgG isotype control via one-way ANOVA with Dunnett’s post-test. (**E**) Fluo-4 Ca^2+^ trace (average of n = 3 experiments) showing response to 50 µM histamine inhibited by H1 antagonist cetirizine. The time of addition of histamine ± cetirizine is denoted by the arrow. (**F**) Immunofluorescence of Akt and eNOS in M0 macrophages. The nuclear DAPI stain is shown in yellow. The scale bar is 5 µm. (**G**) Isotype control (rabbit and goat serum) staining. All images are representative of images from cells from 3 independent donors collected and imaged on different days. The scale bar is 5 µm.

We examined the expression of the three Akt family isoforms in the Database of Immune Cell Expression (DICE) quantitative trait loci and epigenomics database [90] and the Gene Expression Omnibus data set GSE122597, viewed via The Immunological Genome Project (ImmGen; [91]). We noted that Akt1 was the most highly expressed isoform in monocytes (Figure 1A) and peritoneal macrophages (Figure 1B). We confirmed this via performing qPCR on the human monocytes and derived macrophages used in this study (Figure 1C). The identity of macrophages was validated based on the expression of CD14 and CD68 via imaging cytometry (Figure 1D), as described previously [17], as well as histamine-induced calcium responses blocked by an H1 antagonist (Figure 1E) [92]. The immunofluorescence of Akt in macrophages overlapped closely with that of eNOS in some cell regions (Figure 1F,G).

**Figure 2:**
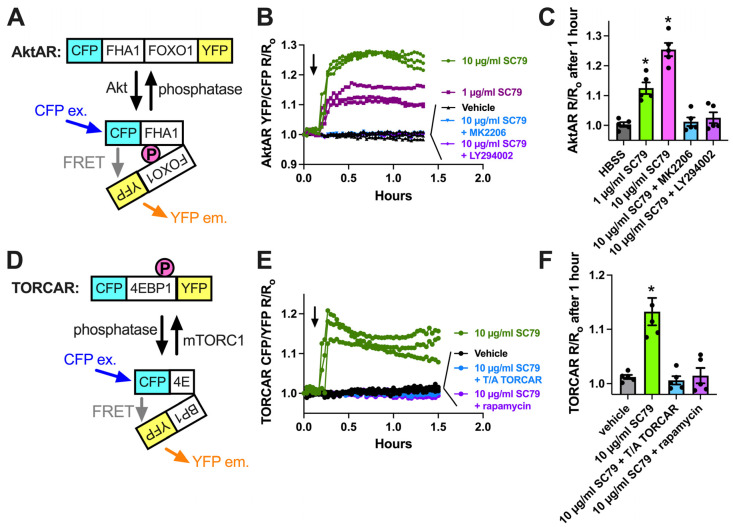
Visualization of SC79 Akt activation in M0 macrophages using fluorescent biosensors. (**A**) Schematic of the AktAR biosensor. Cerulean (cyan fluorescent protein (CFP) variant) and circularly permutated (cp) Venus (YFP variant) surround a forkhead-associated domain (FHA1)-phosphorylated amino acid binding domain and FOXO1 Akt substrate sequence. Akt phosphorylation causes a change in conformation, and a closer proximity of CFP and YFP increases FRET (an increase in the YFP/CFP emission ratio). (**B**) Representative traces from single experiments of AktAR2 YFP/CFP ratio changes in response to 1–10 µM SC79 ± MK2206 or LY294002. Time of addition of the indicated drugs is denoted by the arrow. (**C**) Bar graph of the same responses as in B from 4 independent experiments from different donors per condition. (**D**) Diagram of the TORCAR biosensor. Phosphorylation of the 4EBP1 motif brings CFP and YFP further apart and decreases FRET (an increased CFP/YFP emission ratio). (**E**) Representative traces from single experiments of TORCAR (or mutated T/A control TORCAR) FRET ratio changes in response to 1–10 µM SC79 ± rapamycin. Time of addition of the indicated drugs is denoted by the arrow. (**F**) Bar graph of the same responses as in (**E**) from 4 independent experiments from different donors per condition. Significance was determined via one-way ANOVA with Dunnett’s post-test, comparing values to the vehicle control; * *p* < 0.05.

We next imaged the ability of SC79 to activate Akt using a ratiometric biosensor, AktAR2 [84] (Figure 2A), in cultured and differentiated human macrophages from healthy apheresis donors, as described [17]. AktAR2 changes in terms of conformation when phosphorylated by Akt, resulting in an increase in FRET from cyan fluorescent protein (CFP) brought into close proximity to yellow fluorescent protein (YFP). SC79 increased the ratio of AktAR YFP/CFP emission with CFP excitation, suggesting an increase in Förster resonance energy transfer (FRET). Traces are shown in Figure 2B, and peak changes are shown in Figure 2C. The ratio changes in response to SC79 were inhibited by MK2206 (10 µg/mL), suggesting a dependence on Akt (Figure 2B,C). Activation of Akt by SC79 is dependent on upstream phosphoinositide 3-kinase (PI3K) [35]. Agreeing with this, AktAR ratio changes were also inhibited by PI3K inhibitor LY294002 (10 µg/mL) (Figure 2C). We observed an increase in CFP/YFP emission with TORCAR [93], a biosensor for mTORC1 that changes conformation to bring YFP and CFP further apart via mTORC1 phosphorylation (Figure 2D,E). This was blocked by the mTOR inhibitor rapamycin (Figure 2E,F). As mTORC1 is downstream of Akt, this further supports the SC79 activation of Akt in macrophages. 

### 3.2. SC79/Akt Activation of NO and Downstream cGMP in Human Macrophages

**Figure 3:**
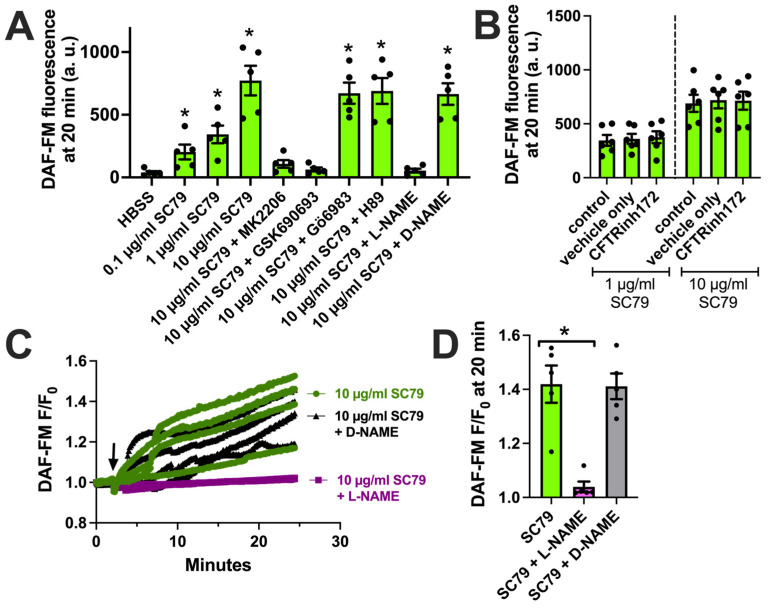
SC79 activates NO production via Akt. (**A**) Bar graph of endpoint DAF-FM fluorescence from 5 independent experiments using macrophages from different donors. Responses were tested with 0.1–10 µg/mL SC79 ± Akt inhibitors MK2206 (10 µg/mL) or GSK690693 (10 µM), PKC inhibitor Gö6983 (10 µM), PKA inhibitor H89 (10 µM), NOS inhibitor L-NAME (10 µM), or inactive D-NAME (10 µM). Significance was determined via one-way ANOVA with Dunnett’s post-test, comparing values to those for HBSS alone; * *p* < 0.05. (**B**) DAF-FM fluorescence data with 1 and 10 µg/mL SC79 ± 10 µM CFTR_inh_172 pretreatment. No significant differences were determined via one-way ANOVA. (**C**) Representative real-time traces of DAF-FM fluorescence, ± L-NAME or D-NAME. Time of addition of the indicated drugs is denoted by the arrow. (**D**) Data from 5 independent experiments done similarly as in (**C**). Significance was determined via one-way ANOVA with Dunnett’s post-test, comparing values to those for SC79 alone; * *p* < 0.05.

We next tested if the activation of Akt by SC79 also resulted in NO production in M0 macrophages, as observed in airway epithelial cells [35,37]. We used DAF-FM, a dye that fluoresces after it covalently reacts with NO, and its reactive degradation products [18]. SC79 (0.1–10 µM) increased DAF-FM fluorescence in macrophages after 30 min of stimulation (Figure 3A). This was blocked by Akt inhibitors MK2206 and GSK690693, but not protein kinase C inhibitor Gö6983 or protein kinase A inhibitor H89 (all at 10 µM; Figure 3A). An SC79-induced DAF-FM fluorescence increase was also blocked by NOS inhibitor N-Nitro-L-arginine methylester (L-NAME) but not the control inactive D-stereoisomer analogue D-NAME (Figure 3A). We previously showed that T2R-induced NO production was decreased in macrophages treated overnight with CFTR inhibitor CFTR_inh_172 (10 µM), a protocol that both blocks CFTR and likely downregulates protein levels [18]. In contrast, we found that SC79-induced NO was not blocked by pretreatment with CFTR_inh_172 (Figure 3B). We confirmed our endpoint measurements by imaging DAF-FM fluorescence changes in real time. We found that 10 µM SC79 induced sustained NO production over ≥20 min that was blocked by L-NAME (Figure 3C,D). Together, these data support that SC79 induces NO production via Akt activation in M0 macrophages. 

**Figure 4:**
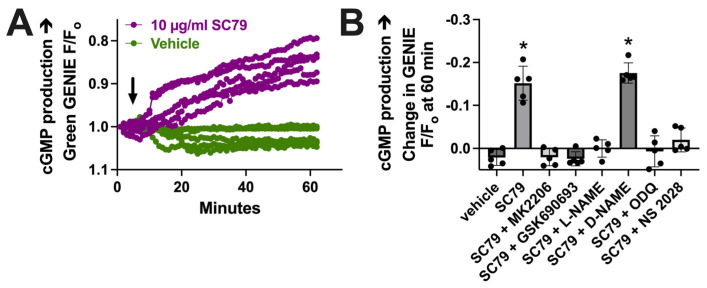
SC79 activates cGMP production downstream of NO. (**A**) Representative traces of cGMP biosensor fluorescence changes with stimulation by 10 µg/mL SC79 vs. the vehicle (0.1% DMSO) only. An upward deflection corresponds to an increase in cGMP levels. Time of addition of the indicated drugs is denoted by the arrow. (**B**) Bar graph of results from independent experiments done similarly as in (**A**). Significance was determined via one-way ANOVA with Dunnett’s post-test, comparing values to those for HBSS plus the vehicle (0.1% DMSO) alone; * *p* < 0.05.

To further test that the data in Figure 3 reflect NO production, we imaged changes in cGMP, which is elevated downstream of the NO activation of soluble guanylyl cyclase. We used a cGMP biosensor delivered to M0 macrophages by BacMam, as previously described [17]. We found that 10 µM SC79 increased cGMP production compared with the vehicle alone (0.1% DMSO; Figure 4A). The change in cGMP was reduced by MK2206, GSK690693, and L-NAME (Figure 4B). It was also blocked by soluble guanylyl cyclase inhibitors 1H-[1,2,4]Oxadiazolo[4,3-a]quinoxalin-1-one (ODQ) and NS 2028 (Figure 4B). Together, these data support that SC79 activates acute NO production, which leads to downstream cGMP production, agreeing with prior data on airway epithelial cells [35,37]. Because we previously showed that acute NO and cGMP production downstream of T2Rs can enhance macrophage phagocytosis [17], we next tested the effects of SC79 on this process.

### 3.3. SC79/Akt Activation of M0 Macrophage Bacterial Phagocytosis

**Figure 5:**
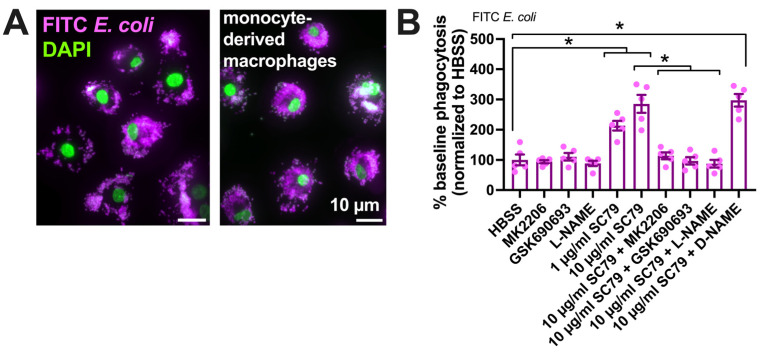
SC79 enhances FITC *E. coli* phagocytosis, likely via Akt and NO signaling. (**A**) Images showing phagocytosis of FITC-labeled *E. coli* (magenta, DAPI nuclear stain in green) in primary human monocyte-derived macrophages, as described [17,19]. (**B**) FITC fluorescence (indicating macrophage phagocytosis) increased with SC79 treatment, which was blocked by Akt inhibitor MK2206 or GSK690693 and NOS inhibitor L-NAME. Significance was determined via one-way ANOVA with Dunnett’s post-test, comparing all values to those of the control; * *p* < 0.05. Data are from 5 independent experiments using cells from 5 donors.

We tested if SC79 enhanced the macrophage phagocytosis of FITC-labeled *Escherichia coli* bioparticles (Figure 5A) on a fluorescence plate reader. SC79 increased macrophage phagocytosis in a manner inhibited by MK2206 and GSK690693 as well as L-NAME (Figure 5B).

**Figure 6:**
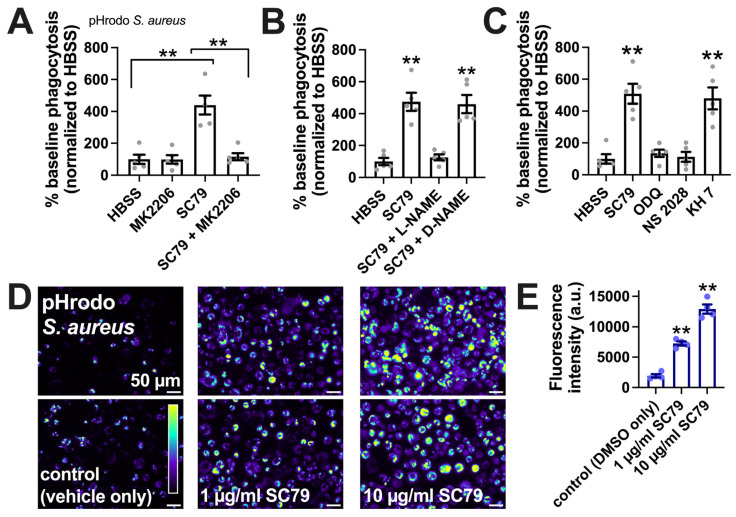
SC79 enhances pHrodo *S. aureus* phagocytosis, likely via Akt and NO signaling. (**A**) The phagocytosis of pHrodo *S. aureus* also increased with 10 µg/mL SC79 and was blocked by MK2206. Note that pHrodo only fluoresces in acidic environments like the phagosome, confirming that internalization reflects phagocytosis. Data were obtained from 5 independent experiments using cells from 5 donors. Significance was determined via one-way ANOVA with Bonferroni’s post-test; ** *p* < 0.01. (**B**) The same type of experiments as in A, but testing SC79 ± L-NAME or D-NAME; significance determined via one-way ANOVA with Dunnett’s post-test, comparing all values to those of the control (HBSS + 0.1% DMSO); ** *p* < 0.01. (**C**) The same type of experiments as in A and B, but testing SC79 ± guanylyl cyclase inhibitor ODQ or NS2028 or adenylyl cyclase inhibitor KH 7 (all at 10 µM); significance determined via one-way ANOVA with Dunnett’s post-test, comparing all values to those of the control (HBSS + 0.1% DMSO); ** *p* < 0.01. (**D**) Micrographs of pHrodo *S. aureus* phagocytosed in macrophages. Top and bottom rows show images from two different donors. (**E**) Quantification of 4 independent experiments, as shown in (**D**), confirming the dose-dependent increase in phagocytosis with SC79; significance was determined via one-way ANOVA with Dunnett’s post-test, comparing all values to those of the control; ** *p* < 0.01.

SC79-stimulated macrophage phagocytosis was confirmed via a plate reader assay of *Staphylococcus aureus* labeled with pH-sensitive dye pHrodo, suggesting increased phagocytosis with SC79 and inhibition by MK2206 (Figure 6A), L-NAME (Figure 6B), and soluble guanylyl cyclase inhibitors 1H-[1,2,4]oxadiazolo[4,3-a]quinoxalin-1-one (ODQ) and NS-2028 (Figure 6C), but not adenylyl cyclase inhibitor KH 7 (Figure 6C). SC79 stimulation of the phagocytosis of *S. aureus* was confirmed via microscopic visualization and quantification, showing dose-dependent effects of SC79 at 1–10 µg/mL (Figure 6D,E).

**Figure 7:**
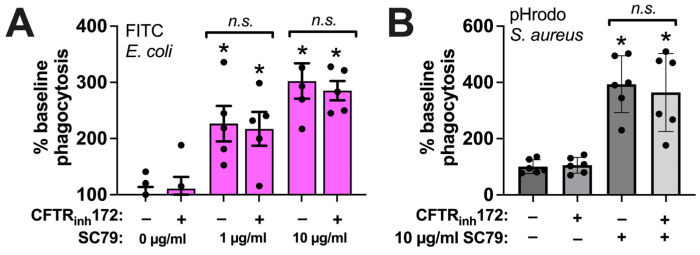
SC79 enhancement of phagocytosis is not altered by CFTR_inh_172. (**A**) The same type of FITC *E. coli* phagocytosis experiments as in Figure 5, testing the SC79 ± CFTR_inh_172 pretreatment. (**B**) The same type of phagocytosis experiments of pHrodo *S. aureus* as in Figure 6, but testing SC79 ± CFTR_inh_172 pretreatment. Significance was determined via one-way ANOVA with Bonferroni’s post-test with paired comparisons; * *p* < 0.05 vs. 0 µg/mL SC79 (HBSS + 0.1% DMSO vehicle control); n.s. means there was no statistical significance between bracketed groups. Data from 5–6 independent experiments per condition with macrophages from different donors.

Phagocytosis of either FITC *E. coli* (Figure 7A) or pHrodo *S. aureus* (Figure 7B) was unaffected by CFTR_inh_172 (10 µM) pretreatment during SC79 treatment (1–10 µg/mL). This suggests that there was no alteration in direct Akt-to-e/nNOS signaling in CF macrophages, despite the alterations in the T2R receptor activation of NOS-driven NO production, that may have occurred with the loss of CFTR function [18,41]. Thus, the direct stimulation of Akt might be a potential target pathway to enhancing NO-driven immune responses in macrophages in the context of CF respiratory infections.

**Figure 8:**
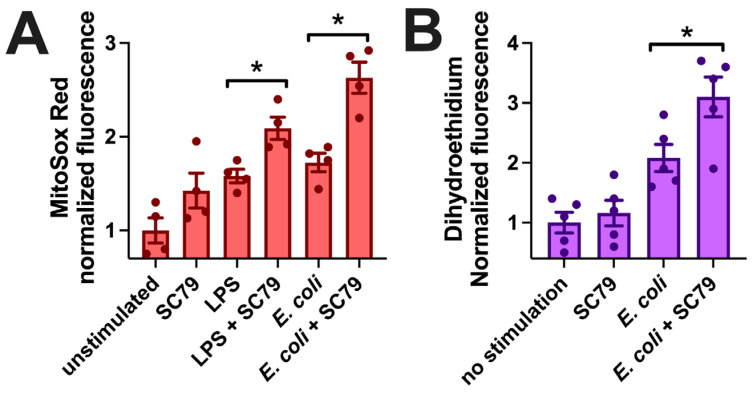
SC79 enhances LPS or bacterial-induced superoxide production. (**A**) Bar graph of macrophage MitoSox Red fluorescence measured on plate reader (396 nm excitation, 610 nm emission) after 60 min stimulation with LPS or *E. coli* ± SC79. (**B**) Bar graph of macrophage dihydroethidium fluorescence (518 nm excitation, 605 nm emission) from experiments similar to those in (**A**). Data from 4–5 independent experiments per condition with macrophages from different donors; significance determined via one-way ANOVA with Bonferroni’s post-test with paired comparisons (±SC79); * *p* < 0.05.

Macrophages can kill phagocytosed bacteria via the production of reactive oxygen species, including superoxide (O_2_•^−^) [94,95]. We measured mitochondrial superoxide production via MitoSox Red, previously used in macrophages to measure ROS production during exposure to LPS, bacteria, or fungi [96,97,98,99]. SC79 did not significantly induce an increase in MitoSox fluorescence, but SC79 (1 µg/mL) significantly enhanced MitoSox fluorescence in the presence of LPS (10 ng/mL) or heat-killed *E. coli* at the same concentration used in the phagocytosis assays above (Figure 8A). We confirmed these results using another superoxide indicator, dihydroethidium (also known as hydroethidine). As with MitoSox, SC79 did not have a significant effect alone but increased dihydroethidium fluorescence in the presence of *E. coli* bioparticles (Figure 8B). Thus, we hypothesize that the increased phagocytosis measured above may translate to increased bacterial killing as well, though this remains to be tested experimentally. All together, these data warrant further investigation into the effects of SC79 on macrophage phagocytosis and bacterial killing.

### 3.4. SC79 May Also Have Anti-Inflammatory Effects, Possibly through Nrf2

Because we previously showed that SC79 reduced IL8 transcription through Nrf2 in airway epithelial cells during Toll-like receptor (TLR) 5 stimulation with *Pseudomonas* flagellin, we tested if SC79 had similar effects in macrophages. Nrf2 was previously shown to reduce pro-inflammatory cytokine secretion in macrophages [100]. Nrf2 in macrophages may be activated downstream of anti-inflammatory metabolite itaconate [101,102], and TPV1 has also been implicated in activating Nrf2 via calcium and CaMKII to inhibit macrophage polarization [103]. The regulation of Nrf2 is complex [104], but it has been shown that PI3K-activated Akt can phosphorylate and inactivate GSK-3β, which reduces the Nrf2 degradation and/or removal of Nrf2 from the nucleus; thus, Akt activation increases the levels of active nuclear-localized Nrf2 [104]. We tested if the SC79 activation of Akt, and possibly Nrf2, could also suppress key inflammatory cytokines activated downstream of the stimulation of TLRs involved in M1 polarization.

**Figure 9:**
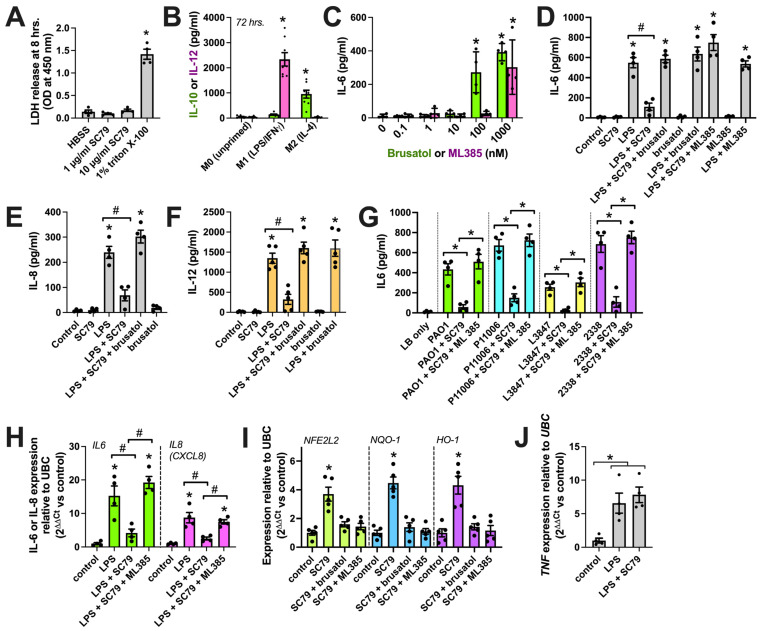
Reduction in macrophage cytokines with SC79. (**A**) LDH release into cell culture media. Staurosporine and triton X-100 were controls used to induce apoptotic death (often followed by secondary necrosis in vitro [107]) and nonspecific lysis, respectively. No LDH was observed with SC79 (one-way ANOVA; Dunnett’s post-test; n = 4 experiments per condition from separate donors); * *p* > 0.05. (**B**) Macrophage IL-12 (M1 marker) or IL-10 (M2 marker) release determined by performing ELISA after 72 h on M1 cocktail (20 ng/mL IFNγ + 100 ng/mL LPS)- or M2-polarizing IL-4 (20 ng/mL). Significance was determined via one-way ANOVA with Bonferroni’s post-test, comparing values with those of M0 (no stimulation); * *p* > 0.05; n = 8 experiments per condition from separate donors. (**C**) Dose response of brusatol or ML385 with IL-6 release. Significance was determined via one-way ANOVA with Bonferroni’s post-test, comparing values with those of the control (no stimulation); * *p* > 0.05; n = 4 experiments from separate donors. (**D**–**F**) Bar graphs of IL-6 (**D**), IL-8 (**E**), or IL-12 (**F**) release with SC79 (10 µg/mL) ± LPS (100 ng/mL) ± 10 nM brusatol or ML385, as indicated. Significance was determined via one-way ANOVA, with Bonferroni’s post-test; * *p* < 0.05 vs. control (media + vehicle) and ^#^ *p* < 0.05 between bracketed columns. n = 4–5 experiments from separate donors. (**G**) The same type of experiments as in D-F but using *P. aeruginosa*-conditioned media ± SC79 ± ML385. Significance was determined via one-way ANOVA with Bonferroni’s post-test; * *p* < 0.05 between bracketed columns. (**H**) IL-6 (green) or IL-8 (magenta) transcript with LPS ± SC79 ± ML385. Significance was tested via one-way ANOVA with Bonferroni’s post-test; * *p* < 0.05 vs. control and ^#^ *p* < 0.05 between bracketed columns; n = 4 experiments from separate donors. (**I**) Nrf2 target transcript levels with SC79 ± brusatol or ML385. Significance was tested via one-way ANOVA with Dunnett’s post-test; * *p* < 0.05 vs. control; n = 4 experiments from separate donors. (**J**) TNF transcript levels with LPS ± SC79. Significance was tested via one-way ANOVA with Dunnett’s post-test; * *p* < 0.05 vs. control; n = 4 experiments from separate donors.

No LDH release was observed from macrophages over 8 h of stimulation with SC79, suggesting no overt toxic effects of SC79 (Figure 9A). We next tested the effects of SC79 against LPS, a Gram-negative bacterial cell wall component and activator of pattern recognition receptors, including TLR4. LPS is part of the classic LPS+IFNγ cocktail for in vitro M1 macrophage polarization that causes an elevation in cytokines like IL12 (Figure 9B). To block Nrf2, we used two distinct pharmacological inhibitors, brusatol [105] and ML385 [106]. We chose concentrations of these inhibitors (10 nM) that did not themselves promote proinflammatory cytokine release via ELISA (the dose response is in Figure 9C, and is also shown in D–F), consistent with our prior observations of airway cells [35]. We found that SC79 reduced IL-6, IL-8, and IL-12 release during stimulation with LPS (Figure 9D–F). This was reversed by ML385 and brusatol (Figure 9D–F). We also found that IL-6 release was reduced with SC79 during exposure to 10% conditioned media from laboratory *P. aeruginosa* (strain PAO1) and clinical isolates (P11006, L3847, 2338) of *P. aeruginosa* from chronic rhinosinusitis patients (Figure 9G). SC79 also reduced *IL6* and *IL8* gene transcription via qPCR during stimulation with LPS, and this was reversed with ML385 (Figure 9H). Further supporting the role of Nrf2, we found that SC79 induced the transcription of itself (*NFE2L2* gene) and Nrf2 target genes *NQO-1* and *HO-1* (Figure 9I). Prior studies suggested that Nrf2 is not able to reduce tumor necrosis factor alpha (TNFα, encoded by the *TNF* gene) transcription with LPS stimulation. We similarly found that *TNF* transcript elevation with LPS was not affected by SC79. All together, these data support the potential anti-inflammatory role of Akt observed via activating Nrf2, likely functioning to inhibit the transcription of a subset of NFκB target genes [100,101].

## 4. Discussion

While at least one Akt isoform is likely expressed in every cell in the human body, the role of Akt in the context of innate immunity is still not fully elucidated [36,108], even despite the knowledge that Akt functions downstream of several pattern recognition receptors like TLRs [43,109]. This may be because studying Akt’s precise role is somewhat complicated by cross-talk between multiple convergent signaling pathways downstream of TLRs and other receptors. However, other studies support a role for Akt in reducing the inflammatory response downstream of the bacterial activation of TLRs in some settings [110,111,112,113]. While PI3K (upstream of AKT) is typically activated by TLRs in many cells [114], there is also precedent to suggest that AKT downregulates TLR-induced signals to NFκB in at least some cells. Notably, TLR4-induced IL-12 expression in human blood-derived monocytes is partly reduced by PI3K, AKT, and JNK [115]. In macrophages, vasoactive intestinal peptide (VIP) reduces TLR4 expression via AKT activation [116]; VIP also downregulates TLR2/4 responses in T regulatory (Treg) cells [117,118], possibly through Akt and/or Nrf2. Quercetin, a T2R14 and T2R39 agonist, was previously shown to reduce the expression of IL6 and to enhance the expression of HO-1 and NQO1 via Akt signaling in macrophages [119]. PI3K and Akt have also been suggested to be essential for macrophage phagocytosis downstream of a variety of receptors [120,121,122,123,124,125,126,127].

Here, we used SC79, a small molecule that interacts with Akt to alter its conformation and allow its phosphorylation by upstream activating kinases [46,47,48,49,50]. This has allowed us to examine the effects of Akt activation independently of other upstream pathways. When Akt is activated downstream of receptors like EGFR, other pathways are activated that might impinge on kinases like Erk in parallel to Akt [128]. When we previously examined the SC79 activation of Akt in nasal and lung epithelial cells [35,37], we found anti-inflammatory effects due to Nrf2 upregulation and translocation to the nucleus [35]. We also showed that the levels of NO produced by nasal epithelial cells during SC79 stimulation can be sufficient to kill clinical strains of bacteria [37].

Here, we show that SC79 also enhances macrophage phagocytosis via Akt and NO signaling. While a previous study showed that SC79 increased macrophage-like mouse Raw264.7 cell phagocytosis [129], our study is the first to show this in human macrophages and tie it to NO. A caveat to this study is that we are working with already-heat-killed bacteria, and while the pHrodo experiments suggest tat the process we are studying is antimicrobial phagocytosis, future studies are needed on live bacteria to look more directly at reactive oxygen species (ROS)-dependent bacterial killing, using antibiotic protection assays and CFU counting, for example [130].

Moreover, we also found that LPS-induced cytokine production is lowered when macrophages are co-stimulated with SC79 via a mechanism that may involve Nrf2, suggesting the inflammation-reducing effects of this molecule’s ability to activate the Akt/Nrf2 pathway. SC79 has also been linked to the activation on Nrf2 in other cell types [47,131,132,133,134], and Nrf2 appears to be involved here based on two different Nrf2 inhibitors. While our data suggest that SC79 can inhibit the onset of an inflammatory response, future experiments are needed to determine the ability of SC79 to reduce cytokine secretion by already activated macrophages.

An important caveat to our study is that we only tested the effects of SC79 in M0 (unprimed/unpolarized) macrophages. It may be that SC79 has other effects on polarized (M1 or M2) macrophages. The role of Akt and its ability to acutely regulate NO production, particularly in M1 macrophages, where inducible NOS (iNOS) is upregulated, and in M2 macrophages, where arginase is upregulated [66,120], must be determined in future studies. Furthermore, the effects of SC79 on macrophage polarization itself must also be determined. Nonetheless, our study represents a demonstration that the Akt pathway can be targeted in naïve M0 macrophages, and this might be used to enhance phagocytosis and/or reduce the transcription of some cytokines. More work, including in vivo testing, is needed to more fully understand if SC79 has potential clinical utility.

It is important to note here that, because Akt plays a central role in metabolism and cell proliferation [12], the PI3K-Akt pathway drives tumorigenesis and/or tumor growth in some cancers [36,128] via mutations in Akt or upstream proteins (e.g., in EGFR or PI3K) that result in aberrant activation of the pathway. These proteins are thus important anti-cancer targets in some cancers [128]. We have not observed increased proliferation with SC79 alone for any of the cells we have tested so far [35,37]. To our knowledge, increased incidence of tumors has not been highlighted in published animal model studies on SC79 [47,48,49,50]. In contrast, SC79 was found to suppress tumor growth by enhancing lymphocyte infiltration into tumors causing cancer cell death [135]. We thus hypothesize that transient pharmacological Akt activation with SC79 may have different effects compared with those of the mutations that arise in cancers. Cancer mutations often lead to prolonged Akt activation. Transient application may avoid unintended proliferative effects. Moreover, cancers cells often have one or more other oncogenic driver mutations in parallel with mutations in the Akt pathway.

Nonetheless, while SC79 is a useful tool for investigating the Akt pathway, we believe it still requires further in vitro and in vivo testing considering its potential to impact cell proliferation. Nonetheless, we believe that our data here and in previous studies [35,37] suggest that acute SC79 application could have both anti-bacterial and anti-inflammatory effects with possibly limited cellular toxicity, at least as shown in our in vitro primary cell models in the setting of short-term (24–48 h) exposure. Limited, targeted delivery of an Akt activator (e.g., as a topical nasal rinse or spray for nasal infections, or as an inhaler for lung infections) may also be a strategy to minimize any undesired effects of activating this pathway systemically.

## 5. Conclusions

This study suggests that a small-molecule activator of Akt like SC79 could be used to modify macrophage phagocytosis and TLR-induced cytokine production in some settings via NO production and Nrf-2, respectively. Further work is needed to understand the effects of targeting the Akt pathway in specific macrophage polarization states as well as in in vivo infection models. This study demonstrates the mechanism of SC79’s effects on unpolarized M0 macrophages, supports an immune role for Akt activation in some settings, and justifies future exploration of SC79 and Akt signaling in models of acute or chronic infection.

## Figures and Tables

**Table 1:** Pharmacological inhibitors and activators used in this study.

Compound	Mechanism/Target	Source (Catalogue Number)
Brusatol	Nrf2 inhibitor	Cayman Chemical (30883)
Cetirizine	H1 histamine receptor antagonist	Cayman Chemical (19686)
CFTR_inh_172	CFTR inhibitor	Cayman Chemical (15545)
cPTIO	Nitric oxide (NO) scavenger	Cayman Chemical (81540)
D-NAME	Inactive analogue of L-NAME	Cayman Chemical (21687)
Gö6983	Protein kinase C (PKC) inhibitor	Cayman Chemical (13311)
GSK690693	Akt inhibitor	Cayman Chemical (16891)
H89	Protein kinase A (PKA) inhibitor	Cayman Chemical (10010556)
KH 7	Soluble adenylyl cyclase (sAC) inhibitor	Cayman Chemical (13243)
L-NAME	Nitric oxide synthase (NOS) inhibitor	Cayman Chemical (80210)
LPS	Toll-like receptor 4 (TLR4) agonist	Millipore Sigma (L9143)
LY294002	PI3K inhibitor	Cayman Chemical (70920)
MK2206	Akt inhibitor	Cayman Chemical (11593)
ML385	Nrf2 inhibitor	Cayman Chemical (21114)
NS 2028	Soluble guanylyl cyclase (sGC) inhibitor	Cayman Chemical (81600)
ODQ	sGC inhibitor	Cayman Chemical (81410)
Rapamycin	mTOR inhibitor	Cayman Chemical (13346)
SC79	Akt activator	Cayman Chemical (14972)
SNAP	NO donor	Cayman Chemical (82250)
U73122	PLC inhibitor	Cayman Chemical (70740)
U73343	Inactive analogue of U73122	Cayman Chemical (17339)

## Data Availability

All data for the study are contained within the manuscript. Raw data points used to generate figures are available upon request.

## References

[1] Ahmad F., Rani A., Alam A., Zarin S., Pandey S., Singh H., Hasnain S.E., Ehtesham N.Z. (2022). Macrophage: A Cell With Many Faces and Functions in Tuberculosis. Front. Immunol..

[2] Pidwill G.R., Gibson J.F., Cole J., Renshaw S.A., Foster S.J. (2020). The Role of Macrophages in Staphylococcus aureus Infection. Front. Immunol..

[3] Reece M.D., Taylor R.R., Song C., Gavegnano C. (2021). Targeting Macrophage Dysregulation for Viral Infections: Novel Targets for Immunomodulators. Front. Immunol..

[4] Dupont M., Sattentau Q.J. (2020). Macrophage Cell-Cell Interactions Promoting HIV-1 Infection. Viruses.

[5] Wang S., Liu R., Yu Q., Dong L., Bi Y., Liu G. (2019). Metabolic reprogramming of macrophages during infections and cancer. Cancer Lett..

[6] Parker D., Prince A. (2011). Innate immunity in the respiratory epithelium. Am. J. Respir. Cell Mol. Biol..

[7] Stevens W.W., Lee R.J., Schleimer R.P., Cohen N.A. (2015). Chronic rhinosinusitis pathogenesis. J. Allergy Clin. Immunol..

[8] Spellberg B., Bartlett J.G., Gilbert D.N. (2013). The future of antibiotics and resistance. N. Engl. J. Med..

[9] Bhattacharyya N., Kepnes L.J. (2008). Assessment of trends in antimicrobial resistance in chronic rhinosinusitis. Ann. Otol. Rhinol. Laryngol..

[10] Genoway K.A., Philpott C.M., Javer A.R. (2011). Pathogen yield and antimicrobial resistance patterns of chronic rhinosinusitis patients presenting to a tertiary rhinology centre. J. Otolaryngol. Head. Neck Surg..

[11] Kingdom T.T., Swain R.E. (2004). The microbiology and antimicrobial resistance patterns in chronic rhinosinusitis. Am. J. Otolaryngol..

[12] Manning B.D., Toker A. (2017). AKT/PKB Signaling: Navigating the Network. Cell.

[13] Mattila J.T., Thomas A.C. (2014). Nitric oxide synthase: Non-canonical expression patterns. Front. Immunol..

[14] Oliveira-Paula G.H., Lacchini R., Tanus-Santos J.E. (2016). Endothelial nitric oxide synthase: From biochemistry and gene structure to clinical implications of NOS3 polymorphisms. Gene.

[15] Sharma N.M., Patel K.P. (2017). Post-translational regulation of neuronal nitric oxide synthase: Implications for sympathoexcitatory states. Expert. Opin. Ther. Targets.

[16] Carey R.M., Lee R.J. (2019). Taste Receptors in Upper Airway Innate Immunity. Nutrients.

[17] Gopallawa I., Freund J.R., Lee R.J. (2021). Bitter taste receptors stimulate phagocytosis in human macrophages through calcium, nitric oxide, and cyclic-GMP signaling. Cell Mol. Life Sci..

[18] Carey R.M., Palmer J.N., Adappa N.D., Lee R.J. (2023). Loss of CFTR function is associated with reduced bitter taste receptor-stimulated nitric oxide innate immune responses in nasal epithelial cells and macrophages. Front. Immunol..

[19] Carey R.M., Hariri B.M., Adappa N.D., Palmer J.N., Lee R.J. (2022). HSP90 Modulates T2R Bitter Taste Receptor Nitric Oxide Production and Innate Immune Responses in Human Airway Epithelial Cells and Macrophages. Cells.

[20] Huang Z., Hoffmann F.W., Fay J.D., Hashimoto A.C., Chapagain M.L., Kaufusi P.H., Hoffmann P.R. (2012). Stimulation of unprimed macrophages with immune complexes triggers a low output of nitric oxide by calcium-dependent neuronal nitric-oxide synthase. J. Biol. Chem..

[21] Reiling N., Ulmer A.J., Duchrow M., Ernst M., Flad H.D., Hauschildt S. (1994). Nitric oxide synthase: mRNA expression of different isoforms in human monocytes/macrophages. Eur. J. Immunol..

[22] Mühl H., Pfeilschifter J. (2003). Endothelial nitric oxide synthase: A determinant of TNFα production by human monocytes/macrophages. Biochem. Biophys. Res. Commun..

[23] Dugas B., Paul-Eugene N., Yamaoka K., Amirand C., Damais C., Kolb J.P. (1995). IL-4 induces cAMP and cGMP in human monocytic cells. Mediat. Inflamm..

[24] Choi H.S., Rai P.R., Chu H.W., Cool C., Chan E.D. (2002). Analysis of nitric oxide synthase and nitrotyrosine expression in human pulmonary tuberculosis. Am. J. Respir. Crit. Care Med..

[25] Mattila J.T., Ojo O.O., Kepka-Lenhart D., Marino S., Kim J.H., Eum S.Y., Via L.E., Barry C.E., Klein E., Kirschner D.E. (2013). Microenvironments in tuberculous granulomas are delineated by distinct populations of macrophage subsets and expression of nitric oxide synthase and arginase isoforms. J. Immunol..

[26] Schmidt H.H., Warner T.D., Nakane M., Forstermann U., Murad F. (1992). Regulation and subcellular location of nitrogen oxide synthases in RAW264.7 macrophages. Mol. Pharmacol..

[27] Connelly L., Jacobs A.T., Palacios-Callender M., Moncada S., Hobbs A.J. (2003). Macrophage endothelial nitric-oxide synthase autoregulates cellular activation and pro-inflammatory protein expression. J. Biol. Chem..

[28] Bogdan C. (2015). Nitric oxide synthase in innate and adaptive immunity: An update. Trends Immunol..

[29] Jorens P.G., Matthys K.E., Bult H. (1995). Modulation of nitric oxide synthase activity in macrophages. Mediat. Inflamm..

[30] Kouakou Y.I., Lee R.J. (2023). Interkingdom Detection of Bacterial Quorum-Sensing Molecules by Mammalian Taste Receptors. Microorganisms.

[31] Dudzinski D.M., Michel T. (2007). Life history of eNOS: Partners and pathways. Cardiovasc. Res..

[32] Forstermann U., Sessa W.C. (2012). Nitric oxide synthases: Regulation and function. Eur. Heart J..

[33] Sanchez-Blazquez P., Rodriguez-Munoz M., Garzon J. (2010). Mu-opioid receptors transiently activate the Akt-nNOS pathway to produce sustained potentiation of PKC-mediated NMDAR-CaMKII signaling. PLoS ONE.

[34] El-Mas M.M., Fan M., Abdel-Rahman A.A. (2009). Facilitation of myocardial PI3K/Akt/nNOS signaling contributes to ethanol-evoked hypotension in female rats. Alcohol. Clin. Exp. Res..

[35] Gopallawa I., Kuek L.E., Adappa N.D., Palmer J.N., Lee R.J. (2021). Small-molecule Akt-activation in airway cells induces NO production and reduces IL-8 transcription through Nrf-2. Respir. Res..

[36] Gopallawa I., Lee R.J. (2020). Targeting the phosphoinositide-3-kinase/protein kinase B pathway in airway innate immunity. World J. Biol. Chem..

[37] Lee R.J., Adappa N.D., Palmer J.N. (2024). Akt activator SC79 stimulates antibacterial nitric oxide generation in human nasal epithelial cells in vitro. Int. Forum Allergy Rhinol..

[38] Paemka L., McCullagh B.N., Abou Alaiwa M.H., Stoltz D.A., Dong Q., Randak C.O., Gray R.D., McCray P.B. (2017). Monocyte derived macrophages from CF pigs exhibit increased inflammatory responses at birth. J. Cyst. Fibros..

[39] Bruscia E.M., Bonfield T.L. (2016). Cystic Fibrosis Lung Immunity: The Role of the Macrophage. J. Innate Immun..

[40] Gao Z., Su X. (2015). CFTR regulates acute inflammatory responses in macrophages. Qjm Int. J. Med..

[41] Totani L., Plebani R., Piccoli A., Di Silvestre S., Lanuti P., Recchiuti A., Cianci E., Dell’Elba G., Sacchetti S., Patruno S. (2017). Mechanisms of endothelial cell dysfunction in cystic fibrosis. Biochim. Biophys. Acta.

[42] Molina S.A., Moriarty H.K., Infield D.T., Imhoff B.R., Vance R.J., Kim A.H., Hansen J.M., Hunt W.R., Koval M., McCarty N.A. (2017). Insulin signaling via the PI3-kinase/Akt pathway regulates airway glucose uptake and barrier function in a CFTR-dependent manner. Am. J. Physiol. Lung Cell Mol. Physiol..

[43] Di Pietro C., Zhang P.X., O’Rourke T.K., Murray T.S., Wang L., Britto C.J., Koff J.L., Krause D.S., Egan M.E., Bruscia E.M. (2017). Ezrin links CFTR to TLR4 signaling to orchestrate anti-bacterial immune response in macrophages. Sci. Rep..

[44] Belchamber K.B.R., Donnelly L.E. (2017). Macrophage Dysfunction in Respiratory Disease. Results Probl. Cell Differ..

[45] Fokkens W.J., Scheeren R.A. (2000). Upper airway defence mechanisms. Paediatr. Respir. Rev..

[46] Jo H., Mondal S., Tan D., Nagata E., Takizawa S., Sharma A.K., Hou Q., Shanmugasundaram K., Prasad A., Tung J.K. (2012). Small molecule-induced cytosolic activation of protein kinase Akt rescues ischemia-elicited neuronal death. Proc. Natl. Acad. Sci. USA.

[47] Li S.T., Chen N.N., Qiao Y.B., Zhu W.L., Ruan J.W., Zhou X.Z. (2016). SC79 rescues osteoblasts from dexamethasone though activating Akt-Nrf2 signaling. Biochem. Biophys. Res. Commun..

[48] Xu Y., Gao Y.W., Yang Y. (2018). SC79 protects dopaminergic neurons from oxidative stress. Oncotarget.

[49] Zhang D., Zhang H., Hao S., Yan H., Zhang Z., Hu Y., Zhuang Z., Li W., Zhou M., Li K. (2016). Akt Specific Activator SC79 Protects against Early Brain Injury following Subarachnoid Hemorrhage. ACS Chem. Neurosci..

[50] Jing Z.T., Liu W., Xue C.R., Wu S.X., Chen W.N., Lin X.J., Lin X. (2019). AKT activator SC79 protects hepatocytes from TNF-alpha-mediated apoptosis and alleviates d-Gal/LPS-induced liver injury. Am. J. Physiol. Gastrointest. Liver Physiol..

[51] Zhang X., Yu Y., Lei H., Cai Y., Shen J., Zhu P., He Q., Zhao M. (2020). The Nrf-2/HO-1 Signaling Axis: A Ray of Hope in Cardiovascular Diseases. Cardiol. Res. Pract..

[52] Zhang X., Ding M., Zhu P., Huang H., Zhuang Q., Shen J., Cai Y., Zhao M., He Q. (2019). New Insights into the Nrf-2/HO-1 Signaling Axis and Its Application in Pediatric Respiratory Diseases. Oxid. Med. Cell Longev..

[53] Zou W., Chen C., Zhong Y., An J., Zhang X., Yu Y., Yu Z., Fu J. (2013). PI3K/Akt pathway mediates Nrf2/ARE activation in human L02 hepatocytes exposed to low-concentration HBCDs. Environ. Sci. Technol..

[54] Salazar M., Rojo A.I., Velasco D., de Sagarra R.M., Cuadrado A. (2006). Glycogen synthase kinase-3beta inhibits the xenobiotic and antioxidant cell response by direct phosphorylation and nuclear exclusion of the transcription factor Nrf2. J. Biol. Chem..

[55] Rada P., Rojo A.I., Chowdhry S., McMahon M., Hayes J.D., Cuadrado A. (2011). SCF/beta-TrCP promotes glycogen synthase kinase 3-dependent degradation of the Nrf2 transcription factor in a Keap1-independent manner. Mol. Cell Biol..

[56] Li R., Jia Z., Zhu H. (2019). Regulation of Nrf2 Signaling. React Oxyg Species.

[57] Rada P., Rojo A.I., Evrard-Todeschi N., Innamorato N.G., Cotte A., Jaworski T., Tobon-Velasco J.C., Devijver H., Garcia-Mayoral M.F., Van Leuven F. (2012). Structural and functional characterization of Nrf2 degradation by the glycogen synthase kinase 3/beta-TrCP axis. Mol. Cell Biol..

[58] Martin D., Rojo A.I., Salinas M., Diaz R., Gallardo G., Alam J., De Galarreta C.M., Cuadrado A. (2004). Regulation of heme oxygenase-1 expression through the phosphatidylinositol 3-kinase/Akt pathway and the Nrf2 transcription factor in response to the antioxidant phytochemical carnosol. J. Biol. Chem..

[59] Lin L., Wu Q., Lu F., Lei J., Zhou Y., Liu Y., Zhu N., Yu Y., Ning Z., She T. (2023). Nrf2 signaling pathway: Current status and potential therapeutic targetable role in human cancers. Front. Oncol..

[60] Casper E. (2023). The crosstalk between Nrf2 and NF-kappaB pathways in coronary artery disease: Can it be regulated by SIRT6?. Life Sci..

[61] Cirone M., D’Orazi G. (2022). NRF2 in Cancer: Cross-Talk with Oncogenic Pathways and Involvement in Gammaherpesvirus-Driven Carcinogenesis. Int. J. Mol. Sci..

[62] van der Horst D., Carter-Timofte M.E., van Grevenynghe J., Laguette N., Dinkova-Kostova A.T., Olagnier D. (2022). Regulation of innate immunity by Nrf2. Curr. Opin. Immunol..

[63] Matsushima K., Yang D., Oppenheim J.J. (2022). Interleukin-8: An evolving chemokine. Cytokine.

[64] Fousek K., Horn L.A., Palena C. (2021). Interleukin-8: A chemokine at the intersection of cancer plasticity, angiogenesis, and immune suppression. Pharmacol. Ther..

[65] Gabarin R.S., Li M., Zimmel P.A., Marshall J.C., Li Y., Zhang H. (2021). Intracellular and Extracellular Lipopolysaccharide Signaling in Sepsis: Avenues for Novel Therapeutic Strategies. J. Innate Immun..

[66] Zamyatina A., Heine H. (2020). Lipopolysaccharide Recognition in the Crossroads of TLR4 and Caspase-4/11 Mediated Inflammatory Pathways. Front. Immunol..

[67] Huszczynski S.M., Lam J.S., Khursigara C.M. (2019). The Role of Pseudomonas aeruginosa Lipopolysaccharide in Bacterial Pathogenesis and Physiology. Pathogens.

[68] Barker J.H., Weiss J.P. (2019). Detecting lipopolysaccharide in the cytosol of mammalian cells: Lessons from MD-2/TLR4. J. Leukoc. Biol..

[69] Valente R.C., Araujo E.G., Rumjanek V.M. (2012). Ouabain inhibits monocyte activation in vitro: Prevention of the proinflammatory mCD14(+)/CD16(+) subset appearance and cell-size progression. J. Exp. Pharmacol..

[70] Lacey D.C., Achuthan A., Fleetwood A.J., Dinh H., Roiniotis J., Scholz G.M., Chang M.W., Beckman S.K., Cook A.D., Hamilton J.A. (2012). Defining GM-CSF- and macrophage-CSF-dependent macrophage responses by in vitro models. J. Immunol..

[71] Ohradanova-Repic A., Machacek C., Fischer M.B., Stockinger H. (2016). Differentiation of human monocytes and derived subsets of macrophages and dendritic cells by the HLDA10 monoclonal antibody panel. Clin. Transl. Immunol..

[72] McMahon D.B., Carey R.M., Kohanski M.A., Tong C.C.L., Papagiannopoulos P., Adappa N.D., Palmer J.N., Lee R.J. (2020). Neuropeptide regulation of secretion and inflammation in human airway gland serous cells. Eur. Respir. J..

[73] McMahon D.B., Carey R.M., Kohanski M.A., Adappa N.D., Palmer J.N., Lee R.J. (2021). PAR-2-activated secretion by airway gland serous cells: Role for CFTR and inhibition by Pseudomonas aeruginosa. Am. J. Physiol. Lung Cell Mol. Physiol..

[74] Kuek L.E., McMahon D.B., Ma R.Z., Miller Z.A., Jolivert J.F., Adappa N.D., Palmer J.N., Lee R.J. (2022). Cilia Stimulatory and Antibacterial Activities of T2R Bitter Taste Receptor Agonist Diphenhydramine: Insights into Repurposing Bitter Drugs for Nasal Infections. Pharmaceuticals.

[75] Carey R.M., Freund J.R., Hariri B.M., Adappa N.D., Palmer J.N., Lee R.J. (2020). Polarization of protease-activated receptor 2 (PAR-2) signaling is altered during airway epithelial remodeling and deciliation. J. Biol. Chem..

[76] Carey R.M., McMahon D.B., Miller Z.A., Kim T., Rajasekaran K., Gopallawa I., Newman J.G., Basu D., Nead K.T., White E.A. (2022). T2R bitter taste receptors regulate apoptosis and may be associated with survival in head and neck squamous cell carcinoma. Mol. Oncol..

[77] Miller Z.A., Mueller A., Kim T., Jolivert J.F., Ma R.Z., Muthuswami S., Park A., McMahon D.B., Nead K.T., Carey R.M. (2023). Lidocaine induces apoptosis in head and neck squamous cell carcinoma through activation of bitter taste receptor T2R14. Cell Rep..

[78] Carey R.M., Adappa N.D., Palmer J.N., Lee R.J. (2021). Neuropeptide Y Reduces Nasal Epithelial T2R Bitter Taste Receptor-Stimulated Nitric Oxide Production. Nutrients.

[79] Schindelin J., Arganda-Carreras I., Frise E., Kaynig V., Longair M., Pietzsch T., Preibisch S., Rueden C., Saalfeld S., Schmid B. (2012). Fiji: An open-source platform for biological-image analysis. Nat. Methods.

[80] Vogel D.Y., Glim J.E., Stavenuiter A.W., Breur M., Heijnen P., Amor S., Dijkstra C.D., Beelen R.H. (2014). Human macrophage polarization in vitro: Maturation and activation methods compared. Immunobiology.

[81] Lathrop S.K., Binder K.A., Starr T., Cooper K.G., Chong A., Carmody A.B., Steele-Mortimer O. (2015). Replication of Salmonella enterica Serovar Typhimurium in Human Monocyte-Derived Macrophages. Infect. Immun..

[82] Tarique A.A., Logan J., Thomas E., Holt P.G., Sly P.D., Fantino E. (2015). Phenotypic, functional, and plasticity features of classical and alternatively activated human macrophages. Am. J. Respir. Cell Mol. Biol..

[83] Vogel D.Y., Vereyken E.J., Glim J.E., Heijnen P.D., Moeton M., van der Valk P., Amor S., Teunissen C.E., van Horssen J., Dijkstra C.D. (2013). Macrophages in inflammatory multiple sclerosis lesions have an intermediate activation status. J. Neuroinflammation.

[84] Gao X., Zhang J. (2008). Spatiotemporal analysis of differential Akt regulation in plasma membrane microdomains. Mol. Biol. Cell.

[85] Freund J.R., Mansfield C.J., Doghramji L.J., Adappa N.D., Palmer J.N., Kennedy D.W., Reed D.R., Jiang P., Lee R.J. (2018). Activation of airway epithelial bitter taste receptors by Pseudomonas aeruginosa quinolones modulates calcium, cyclic-AMP, and nitric oxide signaling. J. Biol. Chem..

[86] Edelstein A., Amodaj N., Hoover K., Vale R., Stuurman N. (2010). Computer control of microscopes using microManager. Curr. Protoc. Mol. Biol..

[87] Mazina O., Reinart-Okugbeni R., Kopanchuk S., Rinken A. (2012). BacMam system for FRET-based cAMP sensor expression in studies of melanocortin MC1 receptor activation. J. Biomol. Screen..

[88] Davenport E.A., Nuthulaganti P., Ames R.S. (2009). BacMam: Versatile gene delivery technology for GPCR assays. Methods Mol. Biol..

[89] Ames R., Fornwald J., Nuthulaganti P., Trill J., Foley J., Buckley P., Kost T., Wu Z., Romanos M. (2004). BacMam recombinant baculoviruses in G protein-coupled receptor drug discovery. Recept. Channels.

[90] Chandra V., Bhattacharyya S., Schmiedel B.J., Madrigal A., Gonzalez-Colin C., Fotsing S., Crinklaw A., Seumois G., Mohammadi P., Kronenberg M. (2021). Promoter-interacting expression quantitative trait loci are enriched for functional genetic variants. Nat. Genet..

[91] Heng T.S., Painter M.W., Immunological Genome Project C. (2008). The Immunological Genome Project: Networks of gene expression in immune cells. Nat. Immunol..

[92] Wang K.Y., Arima N., Higuchi S., Shimajiri S., Tanimoto A., Murata Y., Hamada T., Sasaguri Y. (2000). Switch of histamine receptor expression from H2 to H1 during differentiation of monocytes into macrophages. FEBS Lett..

[93] Zhou X., Li S., Zhang J. (2016). Tracking the Activity of mTORC1 in Living Cells Using Genetically Encoded FRET-based Biosensor TORCAR. Curr. Protoc. Chem. Biol..

[94] Thomas D.C. (2017). The phagocyte respiratory burst: Historical perspectives and recent advances. Immunol. Lett..

[95] Iles K.E., Forman H.J. (2002). Macrophage signaling and respiratory burst. Immunol. Res..

[96] Tur J., Pereira-Lopes S., Vico T., Marin E.A., Munoz J.P., Hernandez-Alvarez M., Cardona P.J., Zorzano A., Lloberas J., Celada A. (2020). Mitofusin 2 in Macrophages Links Mitochondrial ROS Production, Cytokine Release, Phagocytosis, Autophagy, and Bactericidal Activity. Cell Rep..

[97] West A.P., Brodsky I.E., Rahner C., Woo D.K., Erdjument-Bromage H., Tempst P., Walsh M.C., Choi Y., Shadel G.S., Ghosh S. (2011). TLR signalling augments macrophage bactericidal activity through mitochondrial ROS. Nature.

[98] Garcia Del Rio A., Delmiro A., Martin M.A., Cantalapiedra R., Carretero R., Durantez C., Menegotto F., Moran M., Serrano-Lorenzo P., De la Fuente M.A. (2018). The Mitochondrial Isoform of FASTK Modulates Nonopsonic Phagocytosis of Bacteria by Macrophages via Regulation of Respiratory Complex I. J. Immunol..

[99] Hatinguais R., Pradhan A., Brown G.D., Brown A.J.P., Warris A., Shekhova E. (2021). Mitochondrial Reactive Oxygen Species Regulate Immune Responses of Macrophages to Aspergillus fumigatus. Front. Immunol..

[100] Kobayashi E.H., Suzuki T., Funayama R., Nagashima T., Hayashi M., Sekine H., Tanaka N., Moriguchi T., Motohashi H., Nakayama K. (2016). Nrf2 suppresses macrophage inflammatory response by blocking proinflammatory cytokine transcription. Nat. Commun..

[101] Mills E.L., Ryan D.G., Prag H.A., Dikovskaya D., Menon D., Zaslona Z., Jedrychowski M.P., Costa A.S.H., Higgins M., Hams E. (2018). Itaconate is an anti-inflammatory metabolite that activates Nrf2 via alkylation of KEAP1. Nature.

[102] Yin M., Wadhwa R., Marshall J.E., Gillis C.M., Kim R.Y., Dua K., Palsson-McDermott E.M., Fallon P.G., Hansbro P.M., O’Neill L.A.J. (2024). 4-Octyl Itaconate Alleviates Airway Eosinophilic Inflammation by Suppressing Chemokines and Eosinophil Development. J. Immunol..

[103] Lv Z., Xu X., Sun Z., Yang Y.X., Guo H., Li J., Sun K., Wu R., Xu J., Jiang Q. (2021). TRPV1 alleviates osteoarthritis by inhibiting M1 macrophage polarization via Ca(2+)/CaMKII/Nrf2 signaling pathway. Cell Death Dis..

[104] McCord J.M., Gao B., Hybertson B.M. (2023). The Complex Genetic and Epigenetic Regulation of the Nrf2 Pathways: A Review. Antioxidants.

[105] Olayanju A., Copple I.M., Bryan H.K., Edge G.T., Sison R.L., Wong M.W., Lai Z.Q., Lin Z.X., Dunn K., Sanderson C.M. (2015). Brusatol provokes a rapid and transient inhibition of Nrf2 signaling and sensitizes mammalian cells to chemical toxicity-implications for therapeutic targeting of Nrf2. Free Radic. Biol. Med..

[106] Singh A., Venkannagari S., Oh K.H., Zhang Y.Q., Rohde J.M., Liu L., Nimmagadda S., Sudini K., Brimacombe K.R., Gajghate S. (2016). Small Molecule Inhibitor of NRF2 Selectively Intervenes Therapeutic Resistance in KEAP1-Deficient NSCLC Tumors. ACS Chem. Biol..

[107] Silva M.T. (2010). Secondary necrosis: The natural outcome of the complete apoptotic program. FEBS Lett..

[108] Sanchez-Garrido J., Shenoy A.R. (2021). Regulation and repurposing of nutrient sensing and autophagy in innate immunity. Autophagy.

[109] Pourrajab F., Yazdi M.B., Zarch M.B., Zarch M.B., Hekmatimoghaddam S. (2015). Cross talk of the first-line defense TLRs with PI3K/Akt pathway, in preconditioning therapeutic approach. Mol. Cell Ther..

[110] Yu G., Yu H., Yang Q., Wang J., Fan H., Liu G., Wang L., Bello B.K., Zhao P., Zhang H. (2022). Vibrio harveyi infections induce production of proinflammatory cytokines in murine peritoneal macrophages via activation of p38 MAPK and NF-kappaB pathways, but reversed by PI3K/AKT pathways. Dev. Comp. Immunol..

[111] Mao S., Yao J., Zhang T., Zhang X., Tan W., Li C. (2024). Bilobalide attenuates lipopolysaccharide-induced HepG2 cell injury by inhibiting TLR4-NF-kappaB signaling via the PI3K/Akt pathway. Exp. Ther. Med..

[112] Huang C., Sun Y., Qiu X., Huang J., Wang A., Zhang Q., Pang S., Huang Q., Zhou R., Li L. (2022). The Intracellular Interaction of Porcine beta-Defensin 2 with VASH1 Alleviates Inflammation via Akt Signaling Pathway. J. Immunol..

[113] Yang Y., Sun Y., Xu J., Bao K., Luo M., Liu X., Wang Y. (2018). Epithelial Cells Attenuate Toll-Like Receptor-Mediated Inflammatory Responses in Monocyte-Derived Macrophage-Like Cells to Mycobacterium tuberculosis by Modulating the PI3K/Akt/mTOR Signaling Pathway. Mediat. Inflamm..

[114] Troutman T.D., Bazan J.F., Pasare C. (2012). Toll-like receptors, signaling adapters and regulation of the pro-inflammatory response by PI3K. Cell Cycle.

[115] Hildebrand D., Sahr A., Wolfle S.J., Heeg K., Kubatzky K.F. (2012). Regulation of Toll-like receptor 4-mediated immune responses through Pasteurella multocida toxin-induced G protein signalling. Cell Commun. Signal.

[116] Arranz A., Androulidaki A., Zacharioudaki V., Martinez C., Margioris A.N., Gomariz R.P., Tsatsanis C. (2008). Vasoactive intestinal peptide suppresses toll-like receptor 4 expression in macrophages via Akt1 reducing their responsiveness to lipopolysaccharide. Mol. Immunol..

[117] Arranz A., Juarranz Y., Leceta J., Gomariz R.P., Martinez C. (2008). VIP balances innate and adaptive immune responses induced by specific stimulation of TLR2 and TLR4. Peptides.

[118] Arranz A., Gutierrez-Canas I., Carrion M., Juarranz Y., Pablos J.L., Martinez C., Gomariz R.P. (2008). VIP reverses the expression profiling of TLR4-stimulated signaling pathway in rheumatoid arthritis synovial fibroblasts. Mol. Immunol..

[119] Tsai C.F., Chen G.W., Chen Y.C., Shen C.K., Lu D.Y., Yang L.Y., Chen J.H., Yeh W.L. (2021). Regulatory Effects of Quercetin on M1/M2 Macrophage Polarization and Oxidative/Antioxidative Balance. Nutrients.

[120] Vergadi E., Ieronymaki E., Lyroni K., Vaporidi K., Tsatsanis C. (2017). Akt Signaling Pathway in Macrophage Activation and M1/M2 Polarization. J. Immunol..

[121] Lee Y.G., Lee J., Byeon S.E., Yoo D.S., Kim M.H., Lee S.Y., Cho J.Y. (2011). Functional role of Akt in macrophage-mediated innate immunity. Front. Biosci..

[122] Lv Y., Fang L., Ding P., Liu R. (2019). PI3K/Akt-Beclin1 signaling pathway positively regulates phagocytosis and negatively mediates NF-kappaB-dependent inflammation in Staphylococcus aureus-infected macrophages. Biochem. Biophys. Res. Commun..

[123] Ganesan L.P., Wei G., Pengal R.A., Moldovan L., Moldovan N., Ostrowski M.C., Tridandapani S. (2004). The serine/threonine kinase Akt Promotes Fc gamma receptor-mediated phagocytosis in murine macrophages through the activation of p70S6 kinase. J. Biol. Chem..

[124] Jiang M., Chen X.H., Li H., Peng X.X., Peng B. (2023). Exogenous L-Alanine promotes phagocytosis of multidrug-resistant bacterial pathogens. EMBO Rep..

[125] Xin C., Quan H., Kim J.M., Hur Y.H., Shin J.Y., Bae H.B., Choi J.I. (2019). Ginsenoside Rb1 increases macrophage phagocytosis through p38 mitogen-activated protein kinase/Akt pathway. J. Ginseng Res..

[126] Yeo J.C., Wall A.A., Luo L., Stow J.L. (2015). Rab31 and APPL2 enhance FcgammaR-mediated phagocytosis through PI3K/Akt signaling in macrophages. Mol. Biol. Cell.

[127] Zhu M., Li D., Wu Y., Huang X., Wu M. (2014). TREM-2 promotes macrophage-mediated eradication of Pseudomonas aeruginosa via a PI3K/Akt pathway. Scand. J. Immunol..

[128] Hoxhaj G., Manning B.D. (2020). The PI3K-AKT network at the interface of oncogenic signalling and cancer metabolism. Nat. Rev. Cancer.

[129] Chang D., Feng J., Liu H., Liu W., Sharma L., Dela Cruz C.S. (2020). Differential effects of the Akt pathway on the internalization of Klebsiella by lung epithelium and macrophages. Innate Immun..

[130] Kaneko M., Emoto Y., Emoto M. (2016). A Simple, Reproducible, Inexpensive, Yet Old-Fashioned Method for Determining Phagocytic and Bactericidal Activities of Macrophages. Yonsei Med. J..

[131] Gong Y.Q., Huang W., Li K.R., Liu Y.Y., Cao G.F., Cao C., Jiang Q. (2016). SC79 protects retinal pigment epithelium cells from UV radiation via activating Akt-Nrf2 signaling. Oncotarget.

[132] Zhu J.L., Wu Y.Y., Wu D., Luo W.F., Zhang Z.Q., Liu C.F. (2019). SC79, a novel Akt activator, protects dopaminergic neuronal cells from MPP(+) and rotenone. Mol. Cell Biochem..

[133] Zheng K., Zhang Q., Lin G., Li Y., Sheng Z., Wang J., Chen L., Lu H.H. (2017). Activation of Akt by SC79 protects myocardiocytes from oxygen and glucose deprivation (OGD)/re-oxygenation. Oncotarget.

[134] Mavrogonatou E., Angelopoulou M., Rizou S.V., Pratsinis H., Gorgoulis V.G., Kletsas D. (2022). Activation of the JNKs/ATM-p53 axis is indispensable for the cytoprotection of dermal fibroblasts exposed to UVB radiation. Cell Death Dis..

[135] Santinon F., Ezzahra B.F., Bachais M., Sarabia Pacis A., Rudd C.E. (2022). Direct AKT activation in tumor-infiltrating lymphocytes markedly increases interferon-gamma (IFN-gamma) for the regression of tumors resistant to PD-1 checkpoint blockade. Sci. Rep..

